# A Sequential Two-Stage Track-to-Track Association Method in Asynchronous Bearings-Only Sensor Networks for Aerial Targets Surveillance

**DOI:** 10.3390/s19143185

**Published:** 2019-07-19

**Authors:** Yang Yu, Qingyu Hou, Wei Zhang, Jinxiu Zhang

**Affiliations:** School of Astronautics, Harbin Institute of Technology, Harbin 150080, China

**Keywords:** track-to-track association, joint temporal and spatial constraints, asynchronous mono tracks, pairwise fusion model, pairwise similarity model

## Abstract

Successful track-to-track association (TTTA) in a multisensor and multitarget scenario is predicated on a reasonable likelihood function to evaluate the similarity of asynchronous mono tracks. To deal with the lack of synchronous data and prior knowledge of the targets in practical applications, this paper investigates a global optimization method with a novel likelihood function constructed by finite asynchronous measurements with joint temporal and spatial constraints (JTSC). For a scenario with more than two independent sensors, a sequential two-stage strategy is proposed to calculate the similarity of multiple asynchronous mono tracks. For the first stage, based on the temporal features of measurements from different sensors, a pairwise fusion model to estimate the position of the target with two mono tracks is established based on the asynchronous crossing location approach. For the other stage, to evaluate the similarity of the outputs, a pairwise similarity model is constructed by searching for the optimal matching points by setting temporal and spatial constraints. Thus, the likelihood of multiple asynchronous tracks is obtained. Simulations are performed to verify that the proposed method can achieve favorable performance without data-synchronization, and also demonstrate the superiority over the methods based on hinge angle differences (HADs) in some scenarios.

## 1. Introduction

Unmanned aerial vehicles (UAVs) flying in formation are becoming more popular due to the increasing demand of cooperative tasks [1,2]. Infrared sensors, detecting the energy emitted by the targets of interest, are widely used in the surveillance system for maneuvering aerial targets because of advantages such as wide field of view (FOV), high sample rate, and immunity to anti-radar features [3,4,5]. The objective of TTTA is to correctly group the mono tracks originated from the same target at different sensors, thus providing a prerequisite for state estimation and situation awareness. The incorrect association results introduce “ghost” targets, which deteriorate the overall surveillance effect [6,7].

The commonly suggested assumption in the TTTA problem is that all of the measurements are sampled simultaneously, which ignores the inevitable asynchronism. However, this does not hold in real-world applications. A bearings-only sensor network is a complex detection system consisting of multiple optical sensors [8]. Heterogeneous sensors usually have various sampling rates and different detection modes, such as staring and scanning [9,10], in which the measurements are sampled related to the position of the target. For homogeneous sensors, though, it is difficult to eliminate the phase difference because of the independent sampling process. In addition, mono tracks may also exhibit the silent duration phenomenon, where no measurements are present due to communication failure and missing detection in forming a mono track. In conclusion, the measurements integrated from the networks are often not exactly aligned in either temporal or spatial dimensions, even though the mono tracks describe the same movement behavior of the target. The asynchronous feature unfortunately introduces extra temporal biases with the inherent spatial noise, which increases the complexity and difficulty of TTTA. Therefore, it is of great significance to investigate a practical TTTA method for asynchronous mono tracks.

A number of TTTA methods have been proposed for synchronous tracks. For passive bearings-only sensor networks (PBOSNs) without distance measurements in radar networks, angles of arrival are widely used [11,12,13,14]. For two sensors, Roecker [11] and Blackman et al. [12] proposed HADs between two squares constructed by the line of two sensors and one line of sight (LOS) to the target, solving the association problem in sparse target scenarios with Chi-Square test and nearest neighbor (NN) method, respectively. In order to improve the performance in dense targets scenarios, Weidong et al. [13] proposed a global optimization association algorithm employing the statistic based on HADs in previous work [11,12], and analyzed the performance with different target deployments. However, due to the geometric characteristics of the HADs, the aforementioned methods cannot achieve favorable performance for targets with approximate HADs. For example, when two targets are deployed paralleled to the two sensors, the HADs are always nearly zero, even though they are far away from each other.

To further enhance the geometric constraints, some researchers have introduced dynamical models as prior information, estimating the three-dimensional (3D) position of the targets by tracking techniques, e.g., kalman filter (KF) [15], unscented kalman filter (UKF) [16], extended kalman filter (EKF) [17], particle filter (PF) [18,19,20,21], and other methods [22,23,24,25]. Thus, some TTTA methods for radar systems based on angular and distance measurements become feasible. Based on small sensor biases, Tian [26] presented an optimal sub-pattern assignment (OSPA) metric to measure the distance between two reference topologies (RET). Previous studies [27,28] extended the work by taking missing detection and kinematic parameters into consideration. However, the sensor biases were no longer fixed after coordinate system transformation and the association performance was deteriorated by the ambiguity problem. Li [29] and Zhu [30] aimed to simultaneously implement the bias estimation and the association by proposing a joint optimization method, which suffered from a large computational load. In practical applications, however, it is difficult to get dynamic models of targets as prior information because of the various maneuvering capabilities and missions of targets. In addition, simplified models and assumptions limit the tracking performance [15]. 

However, most of the aforementioned references are based on synchronous measurements. For asynchronous tracks, an interpolation is performed in advance, which introduces extra computational load and potentially conflicts with data assumption. In addition, the two-sensors approach is the most widely used platform to illustrate the TTTA method. Considering the complexity of sensor networks, i.e., multiple sensors, a new strategy to reduce the redundant computation of each pairwise sensor is needed.

Given the asynchronous characteristics of measurements in PBOSNs and the difficulty of obtaining the prior dynamic models of maneuvering aerial targets in practical scenarios, a novel TTTA method is investigated based on the finite measurements, i.e., without data-synchronization. Motivated by the TTTA methods in radar systems, the problem in this paper can be dealt with by evaluating the similarity of stereo tracks produced by two mono tracks with the idea of crossing location. Specifically, this paper proposes a novel likelihood function containing a sequential two-stage fusion-based strategy with pairwise mono track fusion and pairwise stereo track similarity evaluation, which can directly deal with the asynchronous measurements. Further, the pairwise fusion model is established to produce a stereo track, estimating the position of the target, with constraints on temporal features of the measurements. Then, a pairwise similarity model for two stereo tracks, i.e., the fusion results, is constructed by setting temporal and spatial constraints. Finally, combing the association strategy for multiple tracks, the overall likelihood of asynchronous mono tracks is obtained. 

The main contributions are as follows:

- A novel likelihood is proposed by an association strategy to couple multiple asynchronous mono tracks and evaluate their similarity in a unified sequential framework without simultaneous measurements.
- A pairwise fusion model using two mono asynchronous to estimate the potential stereo track of the target is presented.
- A pairwise similarity model, based on specific matching data points, is developed to evaluate the similarity of two stereo tracks ranging from 0 to 1.
- A comprehensive association performance evaluation is conducted to illustrate the superiority of the proposed JTSC method over the existing methods based on HADs.

This remainder of this paper is organized as follows. Section 2 converts the TTTA problem into a hypothesis test, providing the global likelihood and the notation used in this paper. Section 3 details the three primary components of the likelihood, including the sequential two-stage fusion-based strategy, the pairwise fusion model, and the pairwise similarity model. In Section 4, simulations are performed to illustrate the performance of the proposed method, and comparisons with HADs-based methods are also done to show the superiority. Finally, some conclusions are drawn in this work.

## 2. Problem Formulation

**Figure 1:**
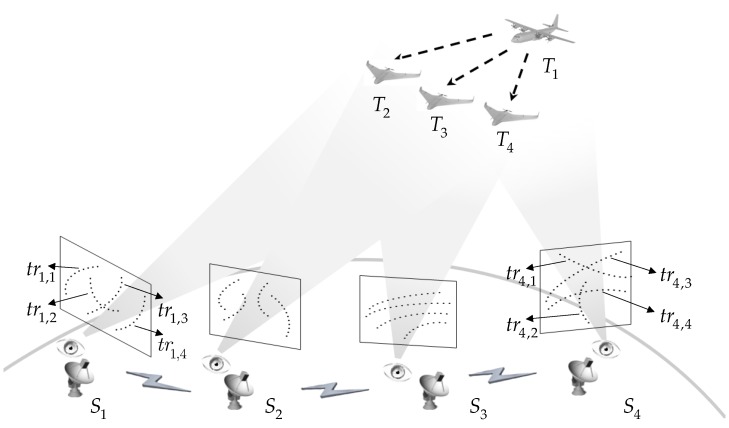
Illustration of multiple aerial targets in passive bearings-only sensor networks.

The information fusion system aggregates the asynchronous mono tracks from each sensor in the PBOSNs to distinguish the corresponding relationship of the tracks and targets. Due to the modern optical remote-sensing techniques, e.g., hyperspectral techniques [31], and advanced image processing techniques, e.g., multi-targets detection and tracking approaches [32,33], the false detections and false tracks are greatly reduced. Thus, this section begins by enumerating some assumptions. Figure 1 shows the scenario of the networks.

- Assumption 1. Every mono track belongs to a certain target. There is no false track or missed track.
- Assumption 2. Every mono track belongs to only one target. The overlapping mono tracks are beyond the scope of this paper.
- Assumption 3. The measurement error and process noise are Gaussian distributed and statistically independent.

Given these assumptions, one is ready to build a mathematical description of mono tracks originated from $N_{T}$ targets at $N_{S}$ ($N_{S}\geq 3$) independent sensors, where $N_{T}$ and $N_{S}$ denote the quantities of the targets and sensors. The jth mono track at ith sensor is denoted as $tr_{i,j}={\left\{<\alpha_{i,j}(t_{n}),\beta_{i,j}(t_{n})>\right\}}_{n=1}^{n_{i,j}}$ for $i=1,\ldots ,N_{S}$, $j=1,\ldots ,N_{T}$. The quantity of the measurements of the mono track is $n_{i,j}$, and $<\alpha_{i,j}(t_{n}),\beta_{i,j}(t_{n})>$ stands for the two measurements, i.e., azimuth angle and elevation angle at sample moment $t_{n}$. 

According to the measurement model discussed in a previous study [34], for a sensor located at $r_{i}(t_{n})={\left[\begin{array}{ccc} x_{i}(t_{n}) & y_{i}(t_{n}) & z_{i}(t_{n}) \end{array}\right]}^{T}$ and a target at $r_{j}(t_{n})={\left[\begin{array}{ccc} x_{j}(t_{n}) & y_{j}(t_{n}) & z_{j}(t_{n}) \end{array}\right]}^{T}$, the two angles are modeled as:

(1) $$\left[\begin{array}{c} \alpha_{i,j}(t_{n})\\ \beta_{i,j}(t_{n}) \end{array}\right]=h(r_{j}(t_{n}),r_{i}(t_{n}))+w_{i,j}(t_{n})$$

where $w_{i,j}(t_{n})$ represents the measurement errors regarded as zero-mean Gaussian noise vector with covariance $R_{i,j}(t_{n})$. The true azimuth angle and elevation angle are detailed as:

(2) $$h(r_{j}(t_{n}),r_{i}(t_{n}))={\left[\begin{array}{cc} \tan^{-1}\frac{y_{j}(t_{n})-y_{i}(t_{n})}{x_{j}(t_{n})-x_{i}(t_{n})} & \sin^{-1}\frac{z_{j}(t_{n})-z_{i}(t_{n})}{\left\|r_{j}(t_{n})-r_{i}(t_{n})\right\|} \end{array}\right]}^{T}$$

**Figure 2:**
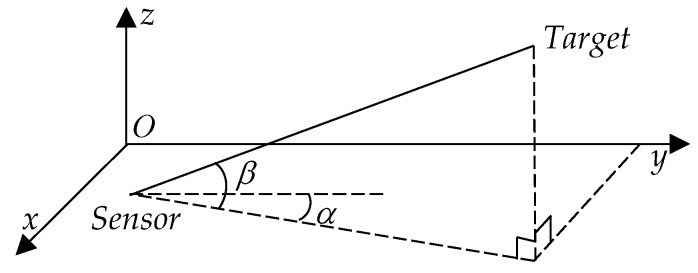
The diagram of the angle measurement.

The geometry including angle measurements is depicted in Figure 2. 

One admissible global association hypothesis ℋ is made up of categorized mono tracks into tuple T, where each tuple stands for the hypothesis that the mono tracks correspond to the same target. Due to the aforementioned assumptions, every valid tuple contains only one mono track from a particular sensor, i.e., an admissible ℋ is a collection of valid tuples that none of the tuples share a mono track and each mono track belongs to a tuple. A valid tuple is denoted as

(3) $$T=\{tr_{i,j}:i=1,\ldots ,N_{S},j=1,\ldots ,N_{T}\}$$

Then, the corresponding tuple likelihood defined in Equation (3) is

(4) $$l(T)=p\{j_{1},\ldots ,j_{N_{S}}|T\}$$

where $j_{i}$ has a one-to-one correspondence to the mono track $tr_{i,j}$ that belongs to tuple T.

The global optimization TTTA method is to determine the most likely global hypothesis ℋ in the set of all valid global hypotheses C. Combing all the tuple likelihoods, the association method can be illustrated as

(5) $$\hat{ℋ}=\arg\underset{ℋ\in C}{\max}\prod_{T\in ℋ}l(T)$$

The global optimization approach is optimal only when each l(T) represents the true likelihood that the probability of the mono tracks originated from the same target. Compared with the multiple frame assignment (MFA) methods for the measurement-to-measurement association problem in previous work [35,36], the two problems share the same mathematical description. Let $c_{j_{1},\ldots ,j_{N_{S}}}=-\ln l(T)$, the TTTA problem described in Equation (5) can be further reformulated by an optimal S-dimensional (S-D) assignment problem as

(6) $$\underset{x_{j_{1},\ldots ,j_{N_{S}}}}{\min}\sum_{j_{1}=1}^{N_{T}}\ldots \sum_{j_{N_{S}}=1}^{N_{T}}x_{j_{1},\ldots ,j_{N_{S}}}c_{j_{1},\ldots ,j_{N_{S}}}$$

subject to

(7) $$\begin{array}{l} \sum_{j_{2}=1}^{N_{T}}\ldots \sum_{j_{N_{S}}=1}^{N_{T}}x_{j_{1},\ldots ,j_{N_{S}}}=1,j_{1}=1,2,\ldots ,N_{T}\\ \sum_{j_{1}=1}^{N_{T}}\ldots \sum_{j_{N_{S}}=1}^{N_{T}}x_{j_{1},\ldots ,j_{N_{S}}}=1,j_{2}=1,2,\ldots ,N_{T}\\ \;\vdots \;\vdots \;\vdots \\ \sum_{j_{1}=1}^{N_{T}}\ldots \sum_{j_{N_{S}-1}=1}^{N_{T}}x_{j_{1},\ldots ,j_{N_{S}}}=1,j_{N_{S}}=1,2,\ldots ,N_{T}\\ x_{j_{1},\ldots ,j_{N_{S}}}\in \{0,1\},\forall j_{1},j_{2},\ldots ,j_{N_{S}} \end{array}$$

where $x_{j_{1},\ldots ,j_{N_{S}}}$ is a binary decision variable, i.e., $x_{j_{1},\ldots ,j_{N_{S}}}=1$ if the $j_{i}$th track at ith sensor is grouped into a tuple. Otherwise, it is 0. The cost function $c_{j_{1},\ldots ,j_{N_{S}}}$ corresponding to the tuple likelihood l(T) in Equation (5), which dominates the performance of association, describes the similarity of the mono tracks. 

So far, the methods to solve the S-D assignment problems have been deeply investigated, e.g., neural networks [37], genetic algorithms [38], and Lagrangian relaxation [36]. The method in a previous study [37] is utilized to solve the problem due to computational efficiency. This paper, however, focuses on the formulation and analysis of suitable approximations of the tuple likelihood, i.e., l(T), in the following section. 

## 3. Tuple Likelihood for Asynchronous Mono Tracks

A unique 3D trajectory is produced along the maneuver of an aerial target. It is difficult to directly measure the differences among mono tracks due to the weak observability. However, the tuple likelihood l(T) can be also explained as the similarity of stereo tracks somehow constructed by the mono tracks. Motivated by the idea of TTTA methods compared with the reference topologies in radar systems, this section presents the three key components of the tuple likelihood for asynchronous mono tracks, listed as: (a) the sequential two-stage fusion-based strategy; (b) the pairwise fusion model; and (c) the pairwise similarity model.

### 3.1. Sequential Two-Stage Fusion-Based Strategy

**Figure 3:**
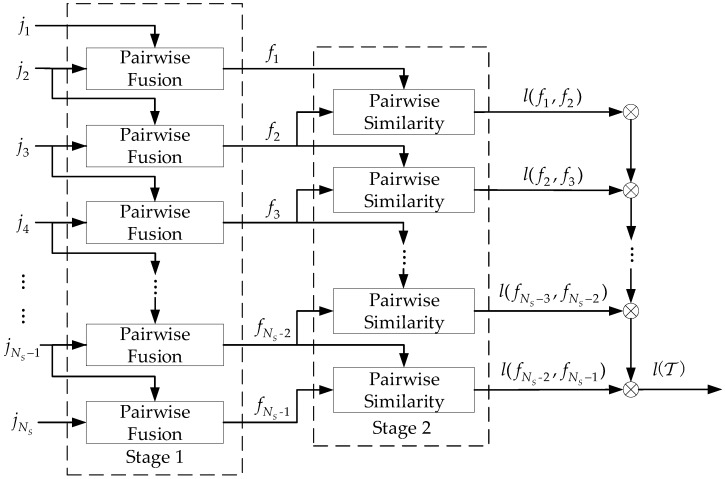
Flow graph of likelihood function calculation strategy for mono-tracks.

Unlike the existing methods for synchronous tracks [7,9,22,25], where the overall likelihood can be based on the comparison with all the simultaneous measurements, the likelihood of asynchronous tracks cannot be obtained in the same way for the lack of unified reference time. To deal with the problem, this subsection introduces a sequential two-stage fusion-based strategy to calculate a tuple likelihood with the flow graph shown in Figure 3.

The blocks in two stages represent the calculation corresponding to pairwise fusion in Section 3.2 and pairwise similarity evaluation in Section 3.3, respectively. In the first stage, the mono tracks $j_{1}$ and $j_{2}$ from the first two sensors are gathered to construct the stereo track $f_{1}$. Then, this stereo track is fixed, and the mono tracks from sensors 2 and 3 are selected. The process continues until tracks from sensors $N_{S}-1$ and $N_{S}$ are collected. The outputs of the first stage are the stereo tracks produced by each two sequential mono tracks, which can be denoted as:

(8) $$ℱ=\{f_{m}:m=1,\ldots ,N_{S}-1\}$$

In the second stage, a similar process is executed by replacing pairwise similarity evaluation with pairwise fusion, and the outputs describing the similarities between the two stereo tracks are denoted as

(9) $$ℒ=\{l(f_{m},f_{m+1}):m=1,\ldots ,N_{S}-2\}$$

Finally, the overall tuple likelihood is the product of the sequential pairwise similarities, so that

(10) $$l(T)=\prod_{m=1}^{N_{S}-2}l(f_{m},f_{m+1})$$

Figure 3 indicates that this strategy couples every three sequential input mono tracks in advance. It is obvious that the tuple likelihood is likely to be larger when T is true because each term in the product of Equation (10) is likely to be larger. A false tuple including four mono tracks can be taken for a counter-example. If only one track $j_{4}$ originates from a different target, the stereo track $f_{3}$ made up of $j_{3}$ and $j_{4}$ is less similar to $f_{2}$. Thus, the tuple likelihood is likely to be smaller with small $l(f_{2},f_{3})$. The two stages, i.e., the pairwise fusion model and the pairwise similarity model, will be introduced in detail in the remainder of this section.

### 3.2. Pairwise Fusion Model

A 3D trajectory can be recognized as a set of 3D positions with time stamps. Motivated by the concept of crossing-location, the position of a target can be estimated with measurements from two sensors. Thus, the nature of pairwise fusion is to utilize the LOS of some specific data points from different tracks, estimating the position sequence of a target by asynchronous crossing-location.

Generally speaking, at the same sampling moment, the two LOS of two independent bearings-only sensors describing the same target should intersect at a certain point, i.e., the real position of the target. In fact, however, it is difficult to find a pair of LOS that satisfy the above conditions because of the different sampling rates and the existence of measurement errors. Instead, a pair of LOS within a certain time interval can be easily found, so it can be assumed that both of them are sampled at the same time. In most cases, these two LOS are skew lines. 

**Figure 4:**
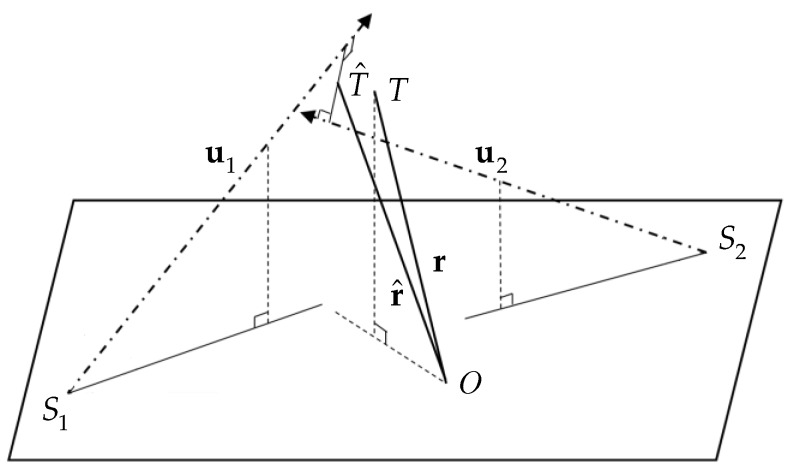
Diagram of asynchronous cross-location by two lines of sight.

Therefore, the problem of estimating the position of a target can be transformed into calculating the midpoint of the common perpendicular constructed by the two LOS with a time interval. The geometric interpretation is that a plane is located through two sensors and shares the same angle with each LOS. Two projections of the LOS on the same plane are produced, and their intersection point is defined as the position by least squares method. The time stamp corresponding to the position estimation is defined as the mid time of the interval. The diagram of asynchronous cross-location by two LOS is shown in Figure 4.

Figure 4 shows a scenario that two bearings-only sensors independently sample the same target T at different instances. Specifically, at sample time $t_{1}$, $s_{1}$ is denoted as the position of the sensor $S_{1}$, and the distance from the sensor to the target T is denoted as $d_{1}$ along a normalized LOS vector $u_{1}$, which can be easily inferred from Equation (1) and Equation (2).

Given a time interval threshold τ, if $\left|t_{1}-t_{2}\right|\leq \tau$, it can be assumed that the two sensors sample the same target simultaneously. An equation can be established as

(11) $$r_{1}=r_{2}=s_{1}+d_{1}u_{1}=s_{2}+d_{2}u_{2}$$

where $r_{1}$ and $r_{2}$ are the positions of the target at different instances. Here, let $U=[\begin{array}{cc} u_{1} & -u_{2} \end{array}]$, $d={\left[\begin{array}{cc} d_{1} & d_{2} \end{array}\right]}^{T}$, and $S=s_{2}-s_{1}$, so Equation (11) can be converted into a matrix form, i.e.,

(12) U⋅d=S

According to the least square method, one can obtain

(13) $$\hat{d}={\left(U^{T}U\right)}^{-1}U^{T}\cdot S$$

After calculating the distance according to $d={\left[\begin{array}{cc} d_{1} & d_{2} \end{array}\right]}^{T}$, the average position of the target can be estimated as:

(14) $$\hat{r}=\frac{1}{2}(s_{1}+d_{1}u_{1}+s_{2}+d_{2}u_{2})$$

The corresponding time stamp of the estimated position is:

(15) $$\hat{t}=\frac{1}{2}(t_{1}+t_{2})$$

The pairwise fusion collects the specific pair measurements satisfying the time threshold to estimate the position of the target by utilizing the method introduced in Equations (14) and (15). The output, i.e., the stereo track, is a time-ordered sequence of discrete data points, expressed by $f_{m}={\left\{r(t_{n})\right\}}_{n=1}^{N_{f_{m}}}$, where $r(t_{n})$ represents the position of the virtual target at the time $t_{n}$, and $N_{f_{m}}$ is the number of data points, i.e., the length of the stereo track. The algorithmic form for pairwise fusion is listed in **Algorithm 1.**

**Algorithm 1:** Pairwise fusion algorithm  **Input**: Two mono tracks: $j_{i}$, $j_{i+1}$; Threshold: temporal threshold τ;  **Output**: Stereo tracks $f_{m}$;

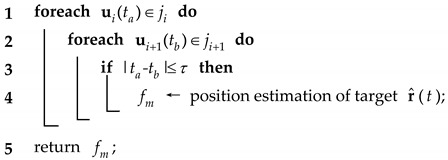



### 3.3. Pairwise Similarity Model

The nature of measuring the similarity between two stereo tracks is to compare the discrete data points. Motivated by the research in previous work [39] dealing with trajectory pattern mining, “clues” extracted from spatially and temporally co-located data points from the observed tracks can be clustered together to analyze the underlying movement behavior if they belong to the same target. 

**Figure 5:**
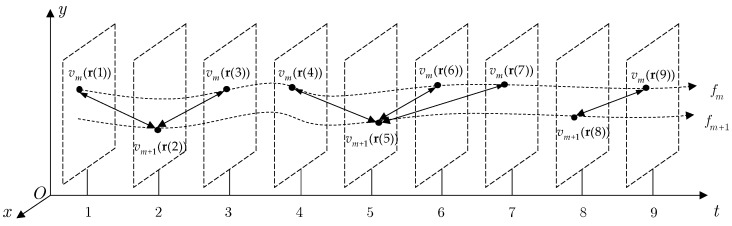
An example to illustrate the pairwise similarity model.

Due to the silent durations in stereo tracks, these “clues” should be identified and utilized in a careful way to reveal the mutual features. Thus, given two stereo tracks, the similarity model tries to identify the “clues” and group as many as possible. A clue-matching scheme and a score of clues to overcome the impact resulting from the temporal and spatial biases are introduced in the following. In Figure 5, an example with two asynchronous stereo tracks, i.e. $f_{m}$ and $f_{m+1}$, is presented to illustrate the pairwise similarity model.

#### 3.3.1. Clues-matching Scheme

If two consecutive stereo tracks describe the movement of the same target, the positions of two data points from different tracks should coincide when they are simultaneously sampled. Due to the inevitable disturbances, such as spatial and temporal biases, temporal shifting, noise, and silent duration [39], it is impossible to make the measurements idealized. However, some data points can be found in pairs from two tracks appearing in a similar area in a certain time delay by setting temporal and spatial tolerance. They can be regarded as “clues” to evaluate the similarity for partial tracks. Here, they are called joint-temporal-spatial matching points (JTSMPs) and defined as follows:

**Definition** **1.**  ***JTSMPs:** Given two tracks $f_{m}$ and $f_{m+1}$, a temporal threshold τ, a spatial threshold ε, and two data points $v_{m}(r(t_{p}))\in f_{m}$, $v_{m+1}(r(t_{q}))\in f_{m+1}$, the two data points can be called JTSMPs if they satisfy the following two criteria:*

(16) $$\begin{array}{l} t(v_{m}(r(t_{p})),v_{m+1}(r(t_{q})))\leq \tau \\ d(v_{m}(r(t_{p})),v_{m+1}(r(t_{q})))\leq \varepsilon \end{array}$$

* where t(⋅,⋅) and d(⋅,⋅) denote the time interval and Euclidean distance between the two points. In Figure 5, each pair of data points connected by a two-way arrow are JTSMPs.*

#### 3.3.2. Score of Clues

A novel scheme is introduced in the aforementioned part to find the “clues”, i.e., JTSMPs. In this part, a decaying function is given to evaluate the strength of the clues containing temporal shifting and spatial biases, i.e., how close the data points are in both temporal and spatial aspects.

**Definition** **2.**  ***Decaying Function:** Given two JTSMPs, i.e., $v_{m}(r(t_{p}))\in f_{m}$ and $v_{m+1}(r(t_{q}))\in f_{m+1}$, a decaying function is defined as*

(17) $$k_{\tau ,\varepsilon }(v_{m}(r(t_{p})),v_{m+1}(r(t_{q})))=1-{\left(\frac{d(v_{m}(r(t_{p})),v_{m+1}(r(t_{q})))}{\varepsilon }\right)}^{\frac{\tau }{t(v_{m}(r(t_{p})),v_{m+1}(r(t_{q})))}}$$

In this function, both the temporal shifting and spatial bias are calculated to evaluate the strength of JTSMPs in a unified framework. The two constants, i.e., temporal threshold τ and spatial threshold ε, give scales to reflect the joint level of the biases and limit the value of this function to a range from 0 to 1. Besides, the decaying function performs a continuous space quantization compared to the discrete space quantization employed in LCSS [40], e.g., the less the difference in time interval and distance is, the larger the value is.

Note that the JTSMPs are extracted by a threshold technique; a data point is likely to have more than one data point satisfying the constraints from the other track. For such a data point, there are several ways to evaluate the clues from this point to the other track. Here, we match this point to the closest point on the other track according to the decaying function.

**Definition** **3.**  ***Score of clues:** Given a data point $v_{m}(r(t_{p}))\in f_{m}$, a set of valid JTSMPs $h_{m+1}\subseteq f_{m+1}$, the score of clues $v_{m}(r(t_{p}))$ to track $f_{m+1}$ is defined as:*

(18) $$s(v_{m}(r(t_{p})),f_{m+1})=\max\{k(v_{m}(r(t_{p})),v_{m+1}(r(t_{q})))|v_{m+1}(r(t_{q}))\in h_{m+1}\}$$

In Figure 5, it is clear that two pairs JTSMPs, i.e., ($v_{m+1}(r(2))$, $v_{m}(r(1))$) and ($v_{m+1}(r(2))$, $v_{m}(r(3))$), share a point $v_{m+1}(r(2))$. Given τ = 2 s, ε = 5 m, the positions of $v_{m+1}(r(2))$, $v_{m}(r(1))$ and $v_{m}(r(3))$ are ${\left[\begin{array}{ccc} 1 & 1 & 1 \end{array}\right]}^{T}$ m, ${\left[\begin{array}{ccc} 2 & 2 & 2 \end{array}\right]}^{T}$ m and ${\left[\begin{array}{ccc} 1 & 1 & 2 \end{array}\right]}^{T}$ m, respectively, and the outputs of the decaying function in Equation (17) are $1-{\left(\sqrt{3}/5\right)}^{2}=0.88$ and $1-{\left(\sqrt{1}/5\right)}^{2}=0.96$. According to Definition 3, the score of $v_{m+1}(r(2))$ is 0.96.

#### 3.3.3. Pairwise Similarity

Considering that a complete track consists of multiple data points and each threshold-satisfied data point has a score to evaluate the similarity of the partial track, the similarity of the two tracks can be represented by the collection of those scores. Therefore, the similarity between two tracks is defined as follows.

**Definition** **4.**  ***Pairwise Similarity:** Given two tracks $f_{m}$ and $f_{m+1}$, the clue-based similarity of $f_{m}$ and $f_{m+1}$ is defined as:*

(19) $$l(f_{m},f_{m+1})=\frac{1}{2N_{f_{m}}}\sum_{v_{m}(r(t_{p}))\in f_{m}}s(v_{m}(r(t_{p})),f_{m+1})+\frac{1}{2N_{f_{m+1}}}\sum_{v_{{}_{m+1}}(r(t_{q}))\in f_{m+1}}s(v_{m+1}(r(t_{q})),f_{m})$$

The similarity is related to both the quantity and the quality of the JTSMPs. Either term is the average score of all points in a track, including those points without JTSMPs, which contributes to distinguishing them from the tracks with few high-scored points, e.g., cross tracks. In addition, a process to average the two terms is designed to balance the similarity due to the nonunique correspondence of the JTSMPs, according to Equation (18). The algorithmic form for the pairwise similarity is listed in Algorithm 2.

**Algorithm 2**: Pairwise Similarity Algorithm  **Input**: 2 Stereo Tracks: $f_{m}$, $f_{m+1}$; Threshold: temporal threshold τ, and spatial threshold ε  **Output**: Pairwise Similarity $l(f_{m},f_{m+1})$

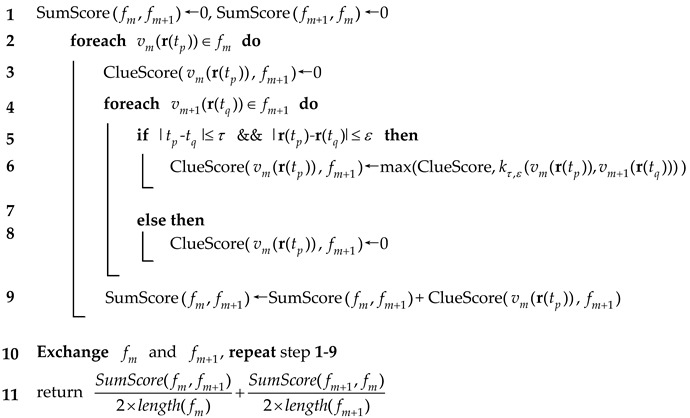



So far, the pairwise similarity model of the two tracks has been introduced. Combining the sequential association strategy in Section 3.1 and the pairwise fusion model in Section 3.2, the likelihood of multiple mono tracks in a tuple can be calculated.

## 4. Simulation Results

**Figure 6:**
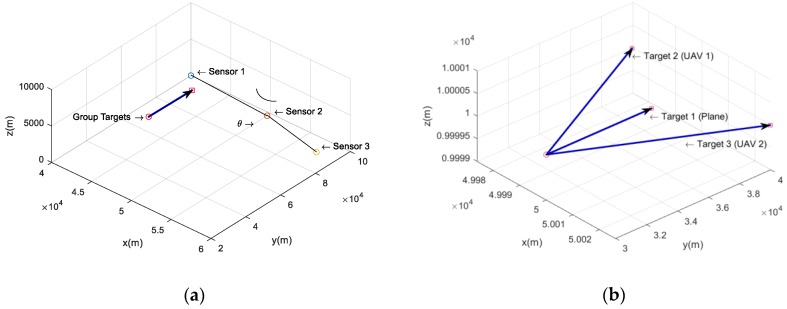
Geometry of the scenario. (**a**) Overall scenario of three deployed sensors and group targets; (**b**) detailed motions of the three aerial targets.

Numerical simulations are performed here to demonstrate the effectiveness of the proposed JTSC method, which focuses on the pairwise fusion model and pairwise similarity model. Then, comparisons with the methods based on HADs are made in terms of the association performance. A scenario consisting of three maneuvering aerial targets and three stable bearings-only sensors in 3D space with a volume of 100,000 m × 100,000 m × 10,000 m is used as an example. For this example, the results are obtained from 100-times Monte Carlo simulation methods. Figure 6a illustrates the geometry of the scenario. 

In this scenario, a plane initially located at ${\left[\begin{array}{ccc} 50000 & 30000 & 10000 \end{array}\right]}^{T}$ m is flying towards +y at a constant speed of 68 m/s. Two UAVs are launched at the beginning of the simulation and are flying at 100 m/s with a deviation of 0.3° relative to +y. The detailed motions of the three aerial targets are shown in Figure 6b. They all fly horizontally at an altitude of 10000 m. Thus, each sensor records three mono tracks corresponding to the plane and the two targets. 

Three sensors are stably deployed on a flat Earth and form the vertices of an isosceles triangle. Each sensor observes the group targets using a staring camera, with different constant sampling rates, from the beginning of the separation. The distances of sensor 1 to sensor 2 and sensor 2 to sensor 3 are equivalent, and the apex angle is initially set as 150°. The detailed parameters for different sensors are listed in Table 1. Considering the failure in forming the correct local mono tracks when the targets are too crowded, an offset time is set to deal with the problem. Here, the offset time is 10 s, which means each mono track begins after 10 s of separation.

### 4.1. Pairwise Fusion Evaluation

This subsection illustrates the effectiveness of the pairwise fusion model introduced in Section 3.2 by comparing all of the potential results with the positions of true targets. Both primary outputs, i.e., the position estimations and the length of the stereo tracks, are analyzed with different parameters of τ, such as 1 s, 2 s, and 3 s. 

**Figure 7:**
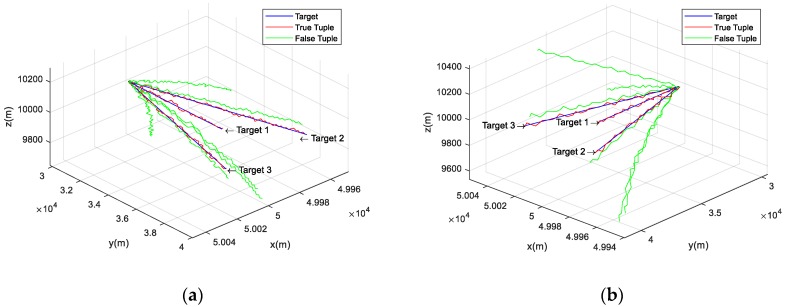
Performance of pairwise fusion model in geometric form. (**a**) All of the potential stereo tracks constructed by sensor 1 and sensor 2. (**b**) All of the potential stereo tracks constructed by sensor 2 and sensor 3.

The input of Section 4.1 and Section 4.2 is a valid tuple T. The tuple T here can be marked as {$j_{1}$, $j_{2}$, $j_{3}$}, in which $j_{i}$ denotes the jth mono track from the ith sensor. To make it easy to understand, the number of the mono track at each sensor corresponds to the target number, e.g., the true tuple of target 1 is {1,1,1}. In addition, if $j_{i}$ is marked as ‘\’, the other two tracks become the inputs of the pairwise fusion model. For example, {2,3,\} means that the second track at sensor 1 and third track at sensor 2 are collected to execute the pairwise fusion. When the simulation time is 100 s, the according geometric performance for τ = 1 s is shown in Figure 7.

In Figure 7, it is obvious that the red stereo tracks are closest to the trajectories of true targets. They are constructed by {1,1,\}, {2,2,\}, and {3,3,\}, i.e., true tuples. In addition, the green lines, derived from the false tuples, are gradually farther from the targets as the tracks grow. This suggests that the pairwise fusion model is capable of fitting the target trajectories and distinguishing them from the false tuple with increasing time, which provides the prerequisites for the pairwise similarity evaluation. The length of the stereo tracks and the average Euclidean distances to the targets are detailed in Table 2.

In Table 2, it is clear that the stereo tracks constructed by true tuples are closest to the corresponding target with a specific τ. The average Euclidean distances of the estimations using the first two sensors and the last two sensors to the ground truth of target 1 can be taken as an example. When τ = 1 s, the distances of true tuples are 17 m and 23 m, respectively, which are much smaller than those of false tuples. In addition, with the increasing parameter τ, two conclusions can be drawn. First, the length of the output is increasing because more constraint-qualified measurements are involved in the pairwise fusion. Second, the larger the τ is, the farther the spatial biases of true tuples are. When τ grows from 1 s to 3 s, there is an increase of 31 m and 44 m by different pair sensors, respectively. This phenomenon results from the position estimations in Equation (11), where the two input LOS are regarded as approximately simultaneous. Thus, larger time intervals between two measurements are equivalent to the larger LOS errors to some extent, which deteriorates the accuracy of the cross-location result.

### 4.2. Pairwise Similarity Evaluation

**Figure 8:**
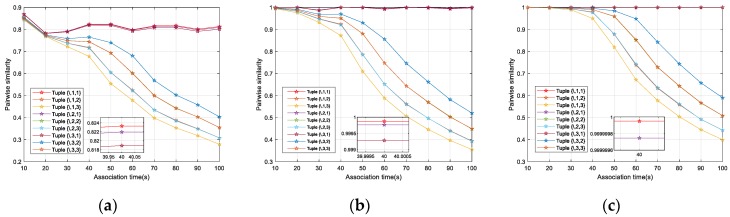
Similarity of stereo tracks derived from true tuples and false tuples versus different parameters τ and ε. (**a**) Similarity for τ = 1 s and ε = 2000 m; (**b**) similarity for τ = 2 s and ε = 2500 m; (**c**) similarity for τ = 3 s and ε = 3000 m.

This subsection illustrates the effectiveness of the pairwise similarity model introduced in Section 3.3 by comparing all the potential stereo tracks with those derived from true tuples for different parameters τ and ε. Figure 8 shows the similarity between stereo tracks by {1,1,\} and all the stereo tracks produced by sensor 2 and sensor 3.

It is obvious that for a specific group of thresholds τ and ε, the pairwise similarity of the stereo tracks derived from true tuple reveals a greater dominance with the association time, i.e., the more data points the stereo tracks contains, the better performance the similarity model shows in discrimination. The similarity of true tuples calculated by Equation (19) gradually gets more significant because more constraint-qualified data points are involved. In addition, these points usually get higher scores of clues in Equation (17) due to smaller spatial biases, which can be obviously observed in Figure 7. For larger threshold parameters, the similarity of all tracks with equivalent lengths maintains a rising trend. However, the similarity of the tracks in true tuples has a disadvantage of significance reduction, e.g., the difference of the similarity decreases from 0.0016 in Figure 8a to 0.0000002 in Figure 8c when the association time is 40 s. The similarities of false tuples benefit more for two reasons. First, more points satisfy the constraints with increasing τ and ε. Second, as is analyzed in Table 2, they enhances the potential to match the false tuple with bare temporal biases and spatial biases according to the maximization in Equation (18). Thus, due to the increase in both quantity and quality of the clues, the similarity of false tuples increases.

### 4.3. Association Performance 

To illustrate the superiority of the proposed method JTSC, the association performance of JTSC is compared with different TTTA methods based on HAD, such as hinge angle difference constraints method (HADC) [13] and nearest neighbor method (NN) [12] for five aspects, i.e., association time, LOS errors, targets density, deployment of sensors, and order of the sequence. 

In principle, the advantage of HADC and NN is that they can deal with the TTTA problem in a scenario where only two sensors exist. JTSC is only available in a network scenario where three sensors are required at least. However, the likelihood in JTSC potentially leads to a better association performance because the likelihood is based on the comparison of the 3D position. It reveals a stronger geometry characteristic than that constructed by angles in HADC and NN. For example, JTSC can potentially overcome the failure for HADC and NN when distributed targets share a small HAD to the sensors.

For the JTSC method developed in this paper, the two thresholds, i.e., the temporal threshold τ and spatial threshold ε, are set in advance. To further illustrate the effectiveness and compare the performance with different parameters, three groups are given, i.e., 1 s and 2000 m, 2 s and 2500 m, and 3 s and 3000 m. As for HADC and NN methods, two operations are performed before the simulation. First, due to the fact that synchronous measurements are the prerequisites for HADC and NN methods, the mono tracks are aligned to the highest sampling rate, i.e., the sampling period is unified as 1.3 s for each sensor. In addition, considering that the methods are based on two sensors, the redundant information derived from different pairwise sensors is employed in the association methods.

To evaluate the association performance of the TTTA methods, the probability of correct association is defined as an evaluation index for the association result as:

(20) $$P_{ca}=\frac{N_{ca}}{N_{T}}$$

where $N_{ca}$ denotes the quantity of true tuples T in a global association hypothesis ℋ, $N_{T}$ denotes the quantity of the targets. In general, the larger the value is, the better association performance it shows.

#### 4.3.1. Simulation for Different Association Times

**Figure 9:**
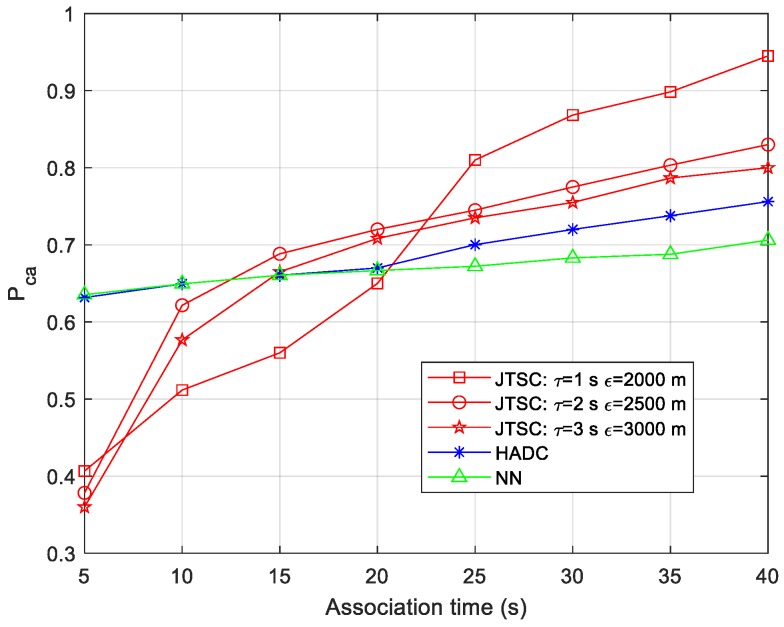
Probability. of correct association vs. different association times.

To analyze the influence of association time, i.e., the length of tracks on the association performance, $P_{ca}$ is investigated when the association time increases from 5 s to 40 s. The results are shown in Figure 9.

With the increasing association time, the $P_{ca}$ of all three methods are always rising. When the association time is less than 10 s, JTSC performs worse than HADC and NN for approximately 0.26. However, it rises tremendously and surpasses the others when the time is longer than 20 s. Meanwhile, smaller threshold parameters lead to larger $P_{ca}$. For example, $P_{ca}$ reaches the top of 0.94 for τ = 1 s and ε = 2000 m, and outperforms those for the other two groups. HADC and NN both grow smoothly from 0.62 to 0.75 and 0.70, respectively; that is to say, HADC increases faster with longer association time. 

The three methods are based on all historical data. With the increasing association time, more valid measurements are available. They help to gradually correct the false association results based on the few measurements greatly affected by random errors, which leads to the improvement of the association performance. For JTSC, due to the asynchronous characteristics, too little time is equivalent to inadequate data points, which fail to reveal the dominant similarity of stereo tracks constructed by true tuples. Larger threshold parameters contribute to supplying more data for similarity evaluation. For sufficient length of the tracks, however, the association performance appears worse with larger parameters because of the reason explained in Section 4.2. HADC and NN are based on HADs. In this scenario, target 2 and target 3 share similar HADs calculated by each of the two sensors, where they are the least positive to sensor 1 and sensor 2. JTSC and NN are based on the global optimization idea and local optimization idea, respectively. The greater redundant HADs calculated by the other two groups of sensors lead to the better association performance for HADC.

#### 4.3.2. Simulation for Different Densities of Targets

**Figure 10:**
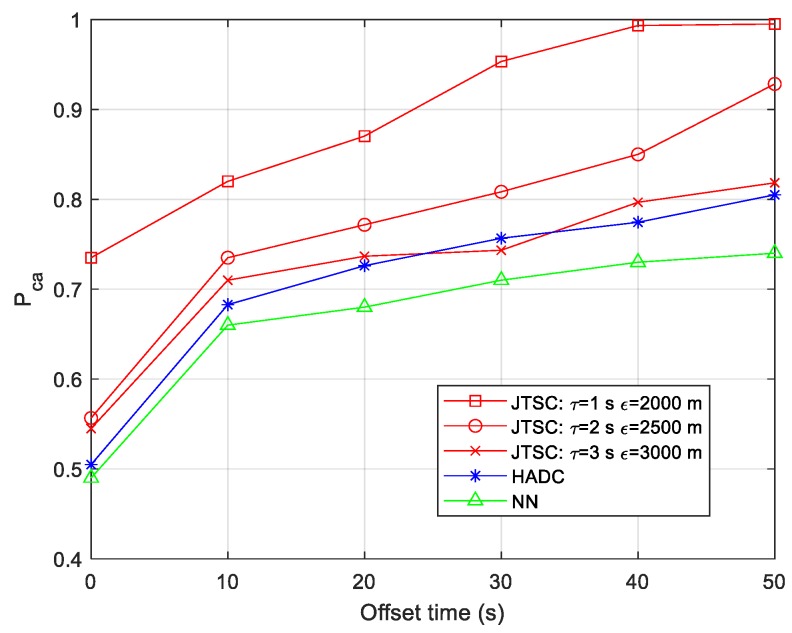
Probability of correct association vs. different offset times.

An offset time is set to determine the start of the mono tracks. In this simulation, the initial relative distances of the targets increase when the offset time grows. Thus, the influence of target density on the association performance can be transformed to analyze the influence of the offset time. Here, the association time is set as 25 s, and $P_{ca}$ is investigated with the offset time ranging from 0 to 50 s. The results are shown in Figure 10.

Figure 10 suggests that with the increasing offset time, all of the methods show better association performance and achieve great improvement when the offset time is 10 s. JTSC overall performs the best with different threshold parameters and grows faster than HADC and NN. Smaller group of threshold parameters always lead to a better performance. Specifically, when the offset time is 40 s, the $P_{ca}$ almost reaches 1 when τ = 1 s and ε = 2000 m, which outperforms those with other parameters for 0.14 and 0.2, respectively. Meanwhile, the NN only reaches 0.73, which is worse than HADC for 0.09. 

HADC and NN are both TTTA methods to find the minimum HADs with targets. The geometric constraints are not strong enough. The approximate HADs are only part of the necessary conditions for homologous tracks, i.e., the mono tracks with the minimum HADs do not necessarily originate from the same target. JTSC is a TTTA method aiming to search for the least differences of the 3D positions of targets, which has more rigorous geometry constraints. In this simulation, the most difficult issue for these methods is to effectively distinguish target 2 from target 3, which always produces approximate HADs by sensor 1 and sensor 2. It is obvious that the increasing offset time, i.e., the density of the targets, contributes little to the HADs, but significantly increases the distance between the two targets. Thus, the JTSC benefits more than the methods based on HAD with larger offset time.

#### 4.3.3. Simulation for Different LOS Errors

**Figure 11:**
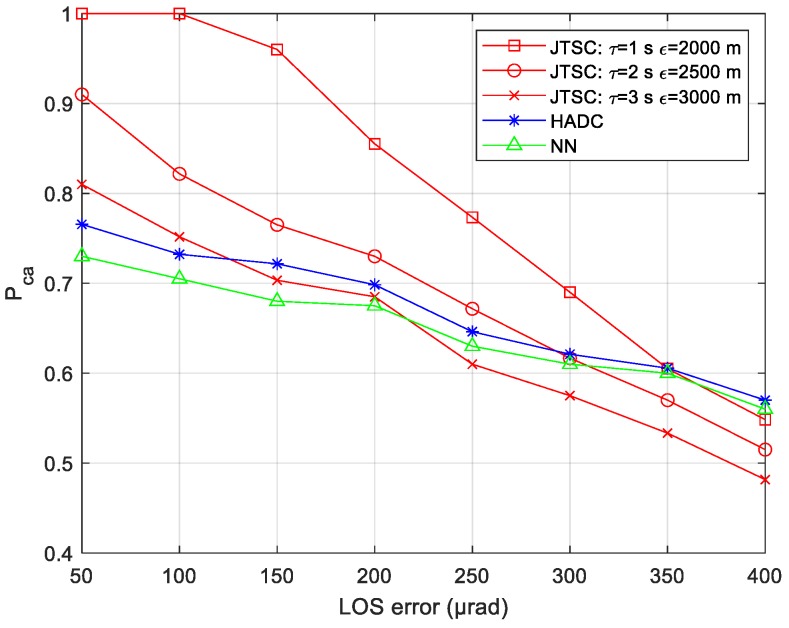
Probability of correct association vs. different LOS errors.

To analyze the influence of the LOS errors on association performance, the association time is fixed at 25 s, and other parameters of the sensors and targets remain identical. This part investigates the $P_{ca}$ when the LOS errors rise from 50 μrad to 400 μrad. The results are shown in Figure 11.

With the increasing LOS errors, all three methods show worse association performance. JTSC performs the steepest reduction, while HADC and NN are relatively smoother. JTSC with the best parameters ebbs from 1 to 0.55, and results are even worse than HADC for 0.02 when the LOS error reaches 400 μrad. HADC creeps down slightly from 0.77 to 0.57, with an overall advantage of 0.02 that is better than NN. It reaches a turning point when the $P_{ca}$ of HADC and NN becomes larger than JTSC when the LOS error is over 350 μrad.

When the LOS error is small enough, considering the stronger geometry constraints, JTSC performs better because it can deal with the failure to distinguish targets with approximate HADs, i.e., target 2 and target 3. JTSC is a two-stage TTTA method consisting of pairwise fusion and pairwise similarity evaluation. The pairwise fusion is based on the idea of asynchronous crossing location, which is sensitive to the LOS error. Larger LOS error deteriorates the accuracy of the position estimations. With the increasing spatial biases, the similarity dominance of the stereo tracks produced by the true tuple is likely to be smaller according to Equation (19), which leads to deterioration of the association performance for JTSC. Thus, its $P_{ca}$ is reduced severely with larger LOS error. HADC and NN, having weaker geometry constraints, are relatively insensitive to the error, which leads to a smooth reduction of the association performance.

#### 4.3.4. Simulation for Different Sensor Deployments

**Figure 12:**
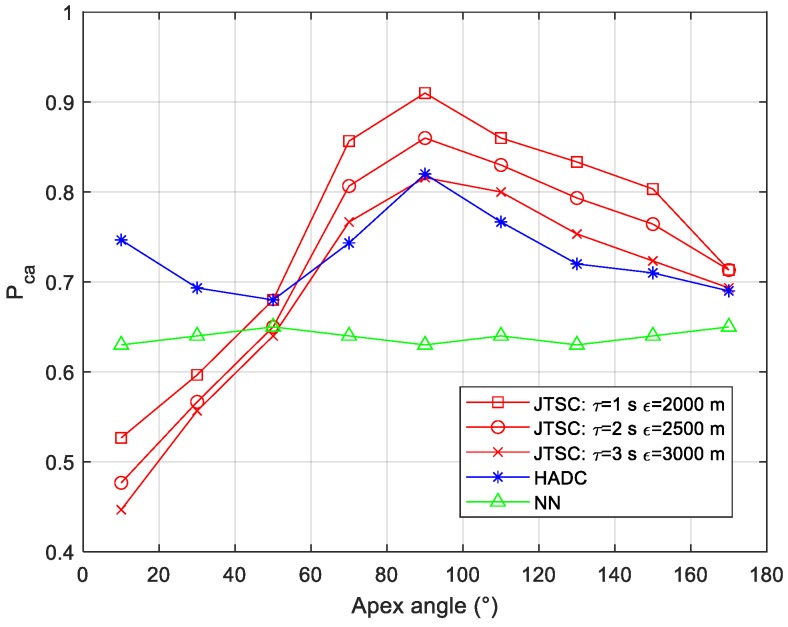
Probability of correct association vs. different apex angle.

To analyze the influence of geometry deployment of the sensors, this subsection analyzes the association performance when the apex angle of the sensors triangle increases from 10° to 170°. Figure 12 shows the results.

Figure 12 shows that NN has the most stable association performance at approximately 0.63, which is always worse than HADC and JTSC with an apex angle larger than 50°. The $P_{ca}$ of HADC reaches two tops of 0.74 and 0.81 when the angle is 10° and 90°, respectively. JTSC is the most sensitive to the angle among the three methods. It rises dramatically when the angle grows from 10° to 90° and subsides moderately otherwise. The fierce increasing trend makes it outperform HADC and NN when the angle is larger than 70° and reaches the summit of 0.91 at 90°. However, JTSC is worse than HADC and NN for 0.23 and 0.11, respectively, with the apex angle of 10°.

For HADC and NN in this simulation, target 2 and target 3 can be effectively distinguished from target 1 by sensor 1 and sensor 2 due to the deployment, which shares a similar conclusion to previous work [13]. Thus, the influence of the apex angle, to a great extent, results in its contribution to the association of the tracks originated from target 2 and target 3. The fluctuating performance of HADC can be explained by two reasons. First, with the increasing apex angle, the HADs of the two targets calculated by sensor 1 and sensor 3 are decreasing, while those by sensor 2 and sensor 3 surge initially and subside afterwards. Second, HADC is based on the global optimization idea with all of the redundant HADs, where the performance is dominated by the best redundant information. Thus, the two tops of the $P_{ca}$ correspond to the angles maximizing the HADs by the aforementioned two sensor pairs. However, NN is restricted by the worst pair of sensors. The performance of JTSC mainly depends on the accuracy of the asynchronous crossing location, which is sensitive to both the angle between the two LOS and the distances between sensors and targets. In addition, when the apex angle is small, all of the potential stereo track performances have greater similarities, especially those from false tuples, because of the approximate deployment of the sensors. This reduces the advantages of the true tuples and deteriorates the association performance as a result. Thus, the JTSC shows poor association performance when the apex angle is smaller than 50°. 

#### 4.3.5. Simulation for Different Order of the Sequence

**Figure 13:**
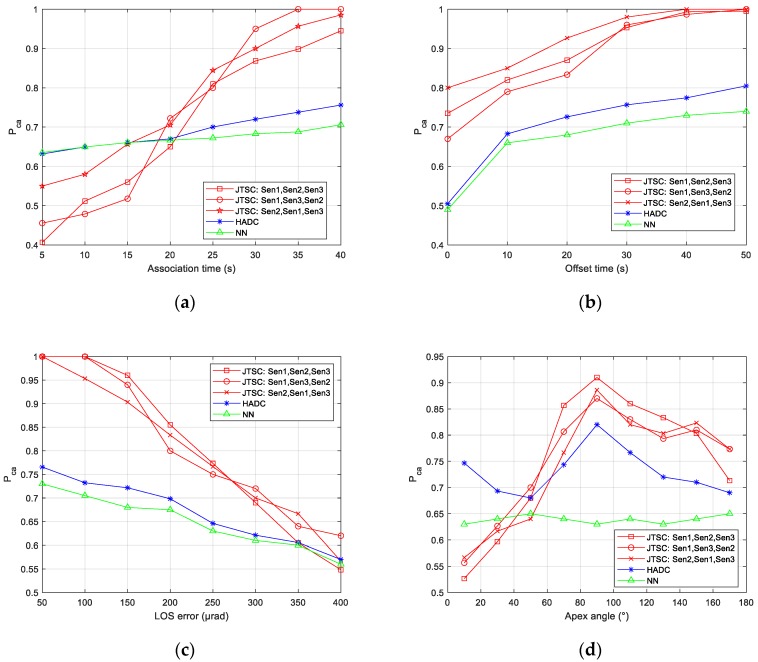
Probability of correct association vs. various conditions in different order. (**a**) Association time; (**b**) offset time; (**c**) LOS error; (**d**) apex angle.

To analyze the influence of order of the sequence presented in Section 3.1, this part discusses the association performance in different orders, i.e., {Sensor 1, Sensor 2, Sensor3}, {Sensor 1, Sensor3, Sensor 2}, and {Sensor 2, Sensor 1, Sensor 3} under various conditions. The thresholds are unified to τ = 1 s and ε = 2000 m. The results are shown in Figure 13.

In the four figures in Figure 13, a common phenomenon appears in different orders of the sequence. In each figure, the association performance shows a robust trend with the increase of various conditions. For example, in Figure 13b, $P_{ca}$ in different orders gradually grows from 0.67, 0.72, 0.80, to approximately 1.0. Specifically, the advantages and disadvantages compared with HADC and NN are always significant. In addition, the curves in JTSC intersect with each other, i.e., none of the orders always performs the best.

The robustness of the JTSC method in different orders relies on the sequential two-stage fusion-based strategy in Section 3.1. It is clear that all of the mono tracks grouped into a tuple have been involved and utilized in this framework, i.e., no information from the input has been ignored or wasted. Besides, the stronger geometry characteristic in the likelihood helps to outperform HADC and NN in principle. However, the slight difference of $P_{ca}$ in different orders is hard to avoid because of the reuse of some tracks, which affects the pairwise fusion and pairwise similarity evaluation. 

In Figure 13d, the similar association performance, when the angle is small, results from different reasons. The failure in the order {Sensor 1, Sensor 2, Sensor3} has been discussed in Section 4.3.4. However, in the other two orders, i.e. {Sensor 1, Sensor 3, Sensor 2} and {Sensor 2, Sensor 1, Sensor 3} it mainly results from the short baseline between Sensor 1 and Sensor 3, which is correlated with the small angle. In most cases, a short baseline limits the performance of positioning by crossing-location methods. The likelihood l(T) of true tuples decreases because it is hard to estimate (recover) the trajectory based on the mono tracks from Sensor 1 and Sensor 3, which consequently leads to the failure.

## 5. Conclusions and Future Work

To deal with the conventional TTTA method’s dependence on data synchronization and prior knowledge of the targets, a novel sequential two-stage association method for aerial target surveillance in asynchronous PBOSNs is investigated in this paper. An effective likelihood function containing three primary components is developed to evaluate the similarity of multiple asynchronous mono tracks by setting joint temporal and spatial constraints to finite measurements. Specifically, an association strategy is designed to couple the multiple mono tracks in a sequential framework, dealing with the failure of simultaneous measurement comparison. A pairwise fusion model is proposed to directly estimate the potential trajectory of the target with asynchronous mono tracks. The pairwise similarity model, by setting temporal and spatial thresholds, is developed to evaluate the similarity of the asynchronous outputs of the fusion model by searching for specific JTSMPs. Numerical simulations are performed to demonstrate the effectiveness of the pairwise fusion model and pairwise similarity model with different parameters. The superiority of the proposed JTSC over HADC and NN has been illustrated and discussed for five aspects, i.e., association time, LOS error, targets density, deployment of the sensors, and order of the sequence. 

Despite the contribution by advanced imaging techniques and detecting techniques, it is still of great importance to take false tracks into consideration because they can hardly be eliminated absolutely. In the future, a modification to normalize the likelihood of consistency and advanced Artificial Intelligence approaches will probably be introduced to improve the effectiveness. It is worth studying the effectiveness and the efficiency can be balanced. 

## Figures and Tables

**Table 1:** Main parameters of sensors in the scenario.

Sensor Number	Position	Sample Period	LOS Error	Offset Time
1	${\left[\begin{array}{ccc} 40000 & 100000 & 0 \end{array}\right]}^{T}$ m	1.3 s	200 μrad	10 s
2	${\left[\begin{array}{ccc} 50000 & 98000 & 0 \end{array}\right]}^{T}$ m	2.3 s	200 μrad	10 s
3	${\left[\begin{array}{ccc} 57660 & 91268 & 0 \end{array}\right]}^{T}$ m	3.0 s	200 μrad	10 s

**Table 2:** Performance of pairwise fusion model with different parameters.

Input Tracks	Euclidean Distances with Different Time Interval Threshold *τ*											
	τ=1 s				τ=2 s				τ=3 s			
	T1	T2	T3	Length	T1	T2	T3	Length	T1	T2	T3	Length
{1,1,\}	17	1594	1594	71	33	1587	1587	137	48	1585	1585	203
{1,2,\}	1665	158	158	71	1658	165	165	137	1656	179	179	203
{1,3,\}	1498	153	153	71	1491	161	161	137	1490	172	172	203
{2,1,\}	136	1554	1554	71	140	1546	1546	137	145	1546	1546	203
{2,2,\}	1592	26	40	71	1587	49	59	137	1584	73	81	203
{2,3,\}	1427	170	172	71	1423	171	173	137	1419	178	181	203
{3,1,\}	243	1377	1376	71	243	1370	1370	137	245	1370	1370	203
{3,2,\}	1758	170	168	71	1751	173	170	137	1749	178	175	203
{3,3,\}	1593	38	24	71	1587	56	45	137	1584	76	67	203
{\,1,1}	23	1577	1577	31	45	1592	1592	60	67	1590	1590	89
{\,1,2}	1788	260	262	31	1798	268	271	60	1802	281	283	89
{\,1,3}	1966	423	425	31	1977	427	428	60	1981	435	437	89
{\,2,1}	155	1643	1643	31	163	1660	1660	60	170	1655	1655	89
{\,2,2}	1582	32	45	31	1591	65	73	60	1596	95	102	89
{\,2,3}	1763	188	190	31	1771	191	193	60	1777	202	205	89
{\,3,1}	312	1844	1843	31	318	1861	1861	60	321	1859	1858	89
{\,3,2}	1400	187	185	31	1408	195	193	60	1413	203	201	89
{\,3,3}	1581	46	34	31	1589	77	68	60	1594	109	102	89
